# High immune responder cows have lower treatment risk and reduced milk loss during highly pathogenic avian influenza H5N1 outbreaks

**DOI:** 10.3168/jdsc.2025-0869

**Published:** 2025-10-10

**Authors:** Shannon C. Beard, Mark E. Carson, Bonnie Mallard, Michael Lohuis, Francesca Malchiodi

**Affiliations:** 1 Semex Alliance, Guelph, ON, Canada, N1H 6J2; 2 Department of Pathobiology, Ontario Veterinary College, University of Guelph, Guelph, ON, Canada, N1G 2W1; 3 Centre for the Genetic Improvement of Livestock, Department of Animal Biosciences, University of Guelph, Guelph, ON, Canada, N1G 2W1

## Abstract

The spillover of highly pathogenic avian influenza (HPAI) hemagglutinin type 5 and neuraminidase type 1 (H5N1) into dairy cattle marks the emergence of a new threat to herd health and production efficiency for North American dairy cattle. To address this challenge, this study evaluated whether genetic selection for improved immune response, using Immunity+ GEBVs, can increase resilience to HPAI H5N1 in commercial dairy herds. Treatment records (n = 15,386) and daily milk yield data (n = 650) were obtained from herd management software from 6 large commercial dairy herds in the United States with confirmed HPAI H5N1 outbreaks. The association between Immunity+ GEBV classes and the likelihood of requiring supportive treatment was assessed using binomial logistic regression, while linear models were used to assess the effects of Immunity+ GEBV class on the length of the milk fluctuation period following treatment and total milk loss during this period. Cows with high Immunity+ GEBVs were 24% less likely to require supportive treatment compared with those with low Immunity+ GEBVs (odds ratio = 0.76; 95% CI: 0.64 to 0.9). High immunity cows also experienced significantly lower milk loss (484.46 kg; 95% CI: 440.61 to 528.30 kg) during the post-treatment fluctuation period compared with low immunity cows (618.10 kg; 95% CI: 561.08 to 675.12 kg). Overall, these findings support the value of selecting for enhanced immune response to improve animal health, reduce production losses, and strengthen economic resilience during disease challenges.

## Highlights

• Supportive treatment is 24% less likely in high versus low Immunity+ cows.
• Post-treatment milk loss was 134 kg lower in high versus low Immunity+ cows.
• Genomic selection for immune response supports herd health and economic resilience.

## Graphical Abstract

**Figure:**
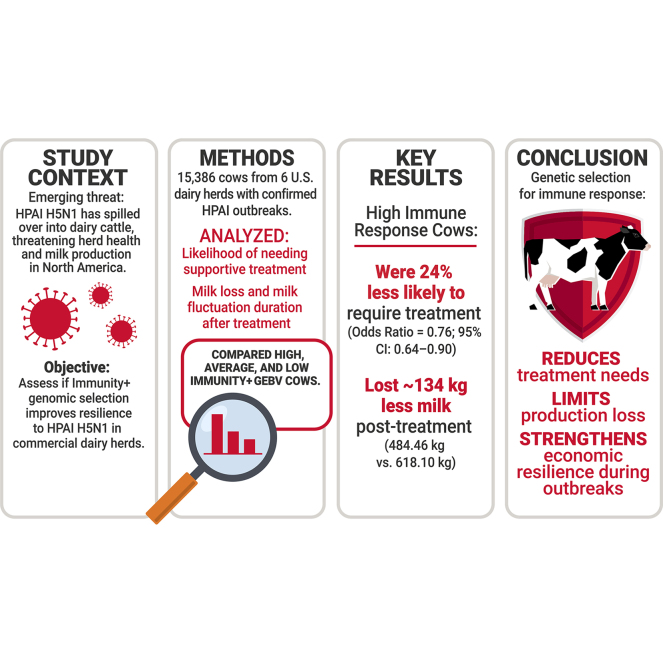
**Summary:** Highly pathogenic avian influenza (HPAI) H5N1 has recently spilled over into US dairy cattle, posing new risks to herd health and milk production. This study evaluated whether genetic selection for improved immune response using Immunity+ genomic estimated breeding values (GEBVs) enhances resilience during HPAI outbreaks. Cows with high Immunity+ GEBVs were 24% less likely to require supportive treatment compared with those with low Immunity+ GEBVs. Following treatment, high Immunity+ GEBV cows lost an average of 134 kg less milk than cows with low Immunity+ GEBVs. These findings demonstrate that genetic selection for immune response can reduce treatment needs, limit production losses, and strengthen economic resilience during infectious disease outbreaks.

Highly pathogenic avian influenza A (**HPAI**) subtype hemagglutinin type 5 and neuraminidase type 1 (**H5N1**) was first identified in poultry in Scotland in 1959 yet did not become widespread in commercial chickens until 1997 (Lycett et al., 2019). After initially circulating in Asia and Europe, the virus reached North America in 2021 (Bevins et al., 2022). Notable for its high mortality rates in wild and domestic birds, HPAI H5N1, especially clade 2.3.4.4b, is also known for its capacity to infect a wide range of species, including domestic cats, swine, marine mammals, and others (Sreenivasan et al., 2024).

In March 2024, the USDA confirmed the first documented spillover of HPAI H5N1 into dairy cattle, following reports of clinical signs and unexplained drops in milk production at a farm in Texas (USDA-APHIS, 2024). As of July 10, 2025, cases have been reported in 1,074 herds across 17 states (USDA-APHIS, 2025), indicating rapid spread; however, this number is likely underestimated due to underreporting and differences in state-level testing requirements. Clinical signs of HPAI H5N1 infection include reduced feed intake and rumination time, respiratory symptoms, lethargy, and milk with abnormal color and consistency (Caserta et al., 2024). Importantly, milk production is also noticeably affected, with average yield losses of 5.7%, translating to an estimated economic impact of approximately $504 per affected cow (Rodriguez et al., 2025). However, individual losses can reach up to 22% (Rodriguez et al., 2025) or as much as $950 per clinically affected cow (Peña-Mosca et al., 2025). The continued spread of HPAI H5N1 in dairy cattle threatens herd health, milk supply stability, and farm profitability, making it critical to identify strategies that can help improve resilience and maintain productivity during outbreaks.

One strategy to reduce the impact of infectious diseases like HPAI H5N1 is to improve herd-level resilience through genetic selection for enhanced immune response. The High Immune Response (**HIR**) technology is a genetic solution designed to enhance broad-based disease resistance in dairy cattle by identifying bulls and cows with superior immune response. This technology directly measures antibody- and cell-mediated immune responses in a reference population, which are then used to calculate GEBVs for immune response in selection candidates. Since 2013, HIR has been licensed by Semex under the tradename Immunity+. In 2022, the Immunity+ index was introduced, which includes the original measures of antibody- and cell-mediated immune responses, as well as nitric oxide and genomic health data from other industry disease evaluations. With a heritability estimate of 30%, Immunity+ offers potential for long-term genetic improvement in disease resilience across commercial dairy herds. Previous studies have shown that cattle identified as high immune responders using Immunity+ have significant reductions in disease, including but not limited to mastitis, pneumonia, and lameness (Wagter et al., 2000; Cartwright et al., 2012; Thompson-Crispi et al., 2013; Larmer and Mallard, 2017).

Building on this, the overall objective of this study was to determine whether a cow's Immunity+ GEBV influences resilience to HPAI H5N1. Resilience was examined through 2 specific objectives. First, the relationship between Immunity+ GEBV and the probability of requiring supportive treatment for HPAI H5N1 in infected herds was evaluated. Second, the association between Immunity+ GEBV and both the duration of the milk fluctuation period following supportive treatment and the milk loss experienced during this period were assessed. Together, these objectives aimed to determine whether a cow's Immunity+ GEBV could serve as an indicator of disease severity and recovery during an HPAI H5N1 outbreak.

For objective 1, treatment records were obtained from on-farm record management software for 15,386 cows from 6 herds in the United States (California and Colorado) with confirmed HPAI H5N1 outbreaks during the period of January to December of 2024. Herd outbreaks were identified through real-time reverse transcriptase PCR testing of bulk tank milk samples collected by certified state samplers (California Department of Food and Agriculture via the California Animal Health and Food Safety Laboratory System or the Colorado Department of Agriculture via the Colorado State University Veterinary Diagnostic Laboratory). Positive results were confirmed by PCR testing at the USDA. Supportive treatment consisted of vitamin B supplementation, delivered either as a bolus or drench. All 6 herds administered vitamin B supplementation as the supportive treatment and the decision to treat was made by the producers without veterinary consultation and was based on clinical signs. Vitamin B was the only supportive treatment used across these herds; no other treatments were reported. Although the specific timing and frequency of administration may have varied slightly between producers, vitamin B was generally used to support recovery in clinically affected animals. Given that it was administered based on observable illness and was used consistently across all study herds, vitamin B treatment serves as a proxy for animals requiring supportive care during the outbreak. Treatment records were consolidated into a single entry per animal, and a binary variable was created to indicate whether the animal had received supportive treatment for HPAI H5N1 during this period. Cows were classified into 3 immune responder groups based on their Immunity+ GEBV. Individuals with a GEBV greater than 1 SD above the population mean were designated as “high” responders, those with a GEBV more than 1 SD below the mean as “low” responders, and those with a GEBV within 1 SD of the mean as “average” responders.

Data analyses were performed using R (R Core Team, 2024; version 8.24.0). The association between Immunity+ GEBV classes and the likelihood of requiring supportive treatment during an HPAI H5N1 outbreak was assessed using binomial logistic regression via the glm() function from the stats package in R, with a logit link. Models were run with Immunity+ GEBV included both as a categorical variable (low, average, high) and as a continuous variable. Treatment status was modeled as

*y_ijkl_* = *μ* + *Imm_i_* + *Par_j_* + *Herd_k_* + *DIM_l_* + *e_ijkl_*,

where *y_ijkl_* is the cow's treatment status during the HPAI H5N1 outbreak (0 = not treated, 1 = required supportive treatment); *μ* is the overall population mean; *Imm_i_* is the fixed effect of Immunity+ GEBV; *Par_j_* is the fixed effect of parity (3 levels: 1, 2, or 3+); *Herd_k_* is the fixed effect of herd (6 levels); *DIM_l_* is the fixed effect of days in milk (5 levels: 5–60, 61–120, 121–180, 181–240, 241–305); and *e_ijkl_* is the random error term. Odds ratios (**OR**) and marginal means of predicted probabilities with 95% CI were estimated to quantify the likelihood of treatment, and group differences were assessed using Tukey-adjusted comparisons to account for multiple testing.

To estimate differences in milk loss associated with HPAI H5N1 infection between Immunity+ GEBVs (objective 2), daily individual milk yield records were extracted for 650 cows treated for HPAI H5N1 for the parity during which the outbreak occurred, across 3 of the 6 herds (daily milk yield data were not available for the remaining 3 herds). Data were filtered to include cows between 5 and 305 DIM and with milk yield between 2.5 and 100 kg per day. Cows were required to have more than 150 records within a lactation, and a minimum of 200 d between the first and last record. Outliers were identified and removed, and missing values were imputed using the tsclean() function from the forecast package in R (Hyndman et al., 2025), which applies a modified Z-score to detect values distant from the median. After cleaning, 119,255 daily milk records for 391 cows remained for further analysis.

An individual lactation curve was estimated for each cow to model expected milk yield in the absence of disturbances. Lactation curves were fitted using fourth-order polynomial quantile regression models to a 0.7 quantile of daily milk yield as a function of DIM:

*y_d_* = *ß*_0_ + *ß*_1_*d* + *ß*_2_*d*^2^ + *ß*_3_*d*^3^ + *ß*_4_*d*^4^ + *ε*,

where *y_d_* is the observed milk yield for an individual cow on DIM *d*; *ß*_0_ is the intercept; *ß*_1_, *ß*_2_, *ß*_3_, and *ß*_4_ are the estimated regression coefficients; and *ε* is the error term. A 0.7 quantile was chosen because it reduces the influence of low milk yield records, providing a lactation curve that more closely reflects expected production in the absence of disturbances. Quantile regression was performed using the poly() function from the quantreg package in R (version 6.1; Koenker, 2025). To avoid distortion associated with recovery-related milk loss, milk yield records from the 35 d following treatment were excluded from model fitting and prediction of the expected lactation curve, which represented the average fluctuation length (**FL**) for all treated cows when an expected lactation curve was fitted without removing any records. A milk fluctuation event was defined as at least 10 consecutive days of negative deviation in milk yield from the expected lactation curve within 30 d post-treatment, including at least one day with milk yield dropping below 90% of expected yield. Fluctuation length was calculated as the total number of days of negative deviation, and milk loss (**ML**, kg) was calculated as the cumulative difference between expected and actual milk yield during the fluctuation.

The effects of Immunity+ GEBV class on FL and ML during the post-treatment milk fluctuation period were evaluated using linear models in R. Models were run with Immunity+ GEBV included both as a categorical variable (low, average, high) and as a continuous variable. Both FL and ML were modeled as

*y_ijkl_* = *μ* + *Imm_i_* + *Par_j_* + *Herd_k_* + *DIM_l_* + *e_ijkl_*,

where *y_ijkl_* is either FL or ML, *μ* is the overall population mean, *Imm_i_* is the fixed effect of Immunity+ GEBV, *Par_j_* is the fixed effect of parity (3 levels: 1, 2, or 3+), *Herd_k_* is the fixed effect of herd (6 levels), *DIM_l_* is the fixed effect of days in milk (5 levels: 5–60, 61–120, 121–180, 181–240, 241–305), and *e_ijkl_* is the random error term. Estimated marginal means and 95% CI were used to compare average ML across Immunity+ GEBV classes, and group differences were assessed using Bonferroni-adjusted comparisons to account for multiple testing.

Summary statistics are presented in Table 1. For the analysis of the effect of Immunity+ GEBV on treatment necessity, Immunity+ GEBVs ranged from 80 to 122, with an overall mean (±SD) of 100.8 ± 4.8. The overall treatment rate across the 6 herds was 22.1%, although this varied considerably between herds, ranging from 13.7% to 52.4%. The incidence rate of HPAI H5N1 is not well documented in the literature; however, reported incidence rates based on clinical signs within individual herds have ranged from 4.75% (Oguzie et al., 2024) to 10% to 15% (Burrough et al., 2024), and up to 32% (Rodriguez et al., 2025). The relatively low average parity (mean ± SD; 1.8 ± 0.9) indicates that many cows were in early lactations, typical of commercial herds where culling rates are high and younger animals comprise a larger proportion of the milking population.

**Table 1:** Summary of descriptive statistics for the traits analyzed

Dataset^1^	n^2^	Trait^3^	Category	Min^4^	Mean^4^	SD	Max^4^	*P*-value^5^
Treatment	15,386	Immunity+ GEBV (categorical)	Low (n = 2,095)	80.0	93.0	2.1	95.0	0.02
			Average (n = 10,713)	96.0	101.0	2.7	105.0	
			High (n = 2,578)	106.0	108.0	2.2	122.0	
		Immunity+ GEBV (continuous)		80.0	100.1	4.8	122.0	<0.01
		DIM		5.0	171.9	99.4	305.0	<0.0001
		Parity		1.0	1.8	0.9	8.0	<0.0001
		Treatment rate (%)		13.7	30.3	15.0	52.4	—
Milk yield	391	Immunity+ GEBV (categorical)	Low (n = 68)	85.0	91.7	2.1	94.0	<0.01
			Average (n = 266)	95.0	99.5	2.8	104.0	
			High (n = 57)	105.0	107.0	2.3	114.0	
		Immunity+ GEBV (continuous)		85.0	99.2	5.2	114.0	<0.001
		DIM		5.0	140.9	77.6	305.0	<0.001
		Parity		1.0	2.7	0.9	5.0	0.09
		Daily milk yield (kg)		0.7	38.1	13.6	88.8	—
		FL (d)		10.0	56.1	23.8	113.0	—
		ML (kg)		12.6	486.7	373.1	2470.6	—

1 Treatment = dataset containing treatment record analysis of the effect of Immunity+ GEBV on HPAI H5N1 treatment necessity; milk yield = dataset containing daily milk yield records for the analysis of the effect of Immunity+ GEBV on ML during the fluctuation period following HPAI H5N1 supportive treatment.

2 n = number of animals.

3 FL = fluctuation length (number of consecutive days of negative deviation in milk yield from the expected lactation curve within 30 d post-treatment for HPAI H5N1, including at least 1 d with milk yield dropping below 90% of expected yield); ML = milk loss (cumulative difference between actual and expected milk yield during the fluctuation after HPAI H5N1 treatment).

4 Min = minimum; mean = arithmetic mean; Max = maximum.

5 *P*-values are from the respective outcome models: treatment probability for the treatment dataset and ML for the milk yield dataset.

The likelihood of requiring treatment for HPAI H5N1 increased with parity. Primiparous cows were significantly less likely to require treatment than cows in their second parity (OR = 0.75, 95% CI: 0.66–0.86; *P* < 0.0001). They were also less likely to require treatment than cows in their third parity (OR = 0.68, 95% CI: 0.59–0.79; *P* < 0.0001). There was no significant difference between parity 2 and 3+. This trend is consistent with findings by Burrough et al. (2024), who also reported increased clinical signs in multiparous cows during an HPAI H5N1 outbreak. Higher parity cows may be more susceptible to severe clinical signs due to greater metabolic demands, higher milk production, or cumulative physiological stress, which can compromise immune function and increase the likelihood of requiring supportive treatment. Cows in early lactation were less likely to require treatment than cows in later lactation. There was no significant difference in treatment probability between cows in 5 to 60 and cows in 61 to 120 DIM, but cows in 5 to 60 DIM were significantly less likely to require treatment than cows in 181 to 240 DIM (OR = 0.69, 95% CI: 0.55–0.88; *P* = 0.0002) and 241 to 305 DIM (OR = 0.73, 95% CI: 0.60–0.88; *P* = 0.0001). Similarly, cows in 61 to 120 DIM were significantly less likely to require treatment than cows in 121 to 180 (OR = 0.67, 95% CI: 0.52–0.87; *P* = 0.0002), 181 to 240 (OR = 0.56, 95% CI: 0.44–0.71; *P* < 0.0001), and 241 to 305 DIM (OR = 0.59, 95% CI: 0.48–0.72; *P* = 0.0001). This pattern also aligns with observations by Burrough et al. (2024), who reported that cows in mid- to late-lactation exhibited more clinical signs than those in early lactation.

Figure 1 illustrates the estimated marginal means for the probability of requiring supportive treatment across Immunity+ GEBV groups, with 95% CI. Cows with high Immunity+ GEBVs had a significantly lower probability of requiring supportive treatment during an HPAI H5N1 outbreak compared with those with low Immunity+ GEBVs (OR = 0.76; 95% CI: 0.64– 0.91; *P* = 0.02), representing a 24% reduction in treatment risk for high Immunity+ cows. When analyzed as a continuous variable, Immunity+ GEBV was also significantly associated with reduced treatment probability (*P* < 0.01). Evidence has indicated that HPAI H5N1 spreads rapidly within dairy herds, likely through contaminated bedding, floors, or mechanically via milking equipment (Caserta et al., 2024). Therefore, it is likely cows with higher Immunity+ GEBVs were exposed to the virus at similar levels as others yet did not develop symptoms severe enough to require treatment. This suggests that these cows may have a greater capacity to mitigate clinical severity following exposure, demonstrating greater resistance to disease.

**Figure 1:**
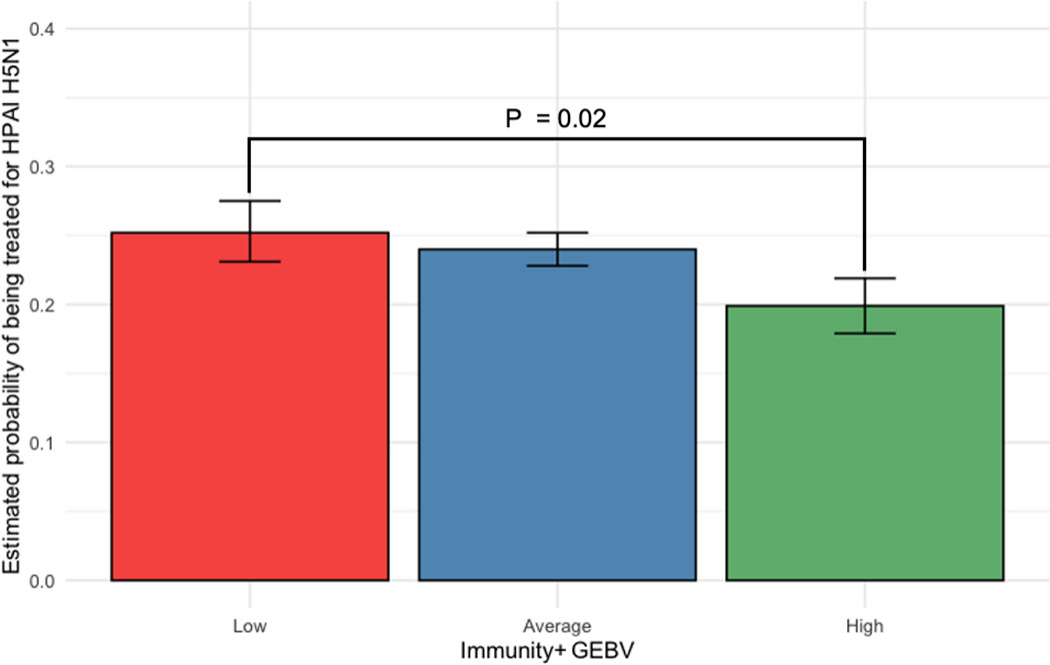
Estimated marginal means (with 95% CI) for the probability of requiring supportive treatment during an outbreak of HPAI H5N1 for Immunity+ GEBV classes (low, n = 2,095; average, n = 10,713; high, n = 2,578).

Supportive treatment in this study was producer-driven and based on observable clinical signs, with vitamin B consistently applied as an intervention across all 6 herds. Treatment was generally given to individually affected animals rather than entire pens, although thresholds for intervention likely varied slightly among herds, and generally no veterinary oversight was involved. For future studies, recording standardized treatment criteria and clinical severity scores would further strengthen interpretation. Despite these limitations, the consistent use of vitamin B as a supportive treatment across herds provides a proxy in this study for producer-perceived need for supportive care.

For the analysis of the effect of Immunity+ GEBV on ML during the fluctuation period following HPAI H5N1 supportive treatment, Immunity+ GEBVs ranged from 85 to 114, with an overall mean (±SD) of 99.2 + 5.2 (Table 1). The average FL was 56 d. There is minimal documentation in the literature of the average duration of the milk fluctuation period following HPAI H5N1 infection, although anecdotal evidence states that production levels may take more than 2 mo to recover (Rodriguez et al., 2024), and epidemiological investigations have shown that cows can maintain decreased milk production for at least 4 wk after showing clinical signs (Caserta et al., 2024). In one study, 5 heifers and 2 lactating cows were experimentally inoculated by an intramammary route with HPAI H5N1, and it was found that milk production remained at 71% to 77% of pre-inoculation production 24 d post-inoculation with the virus (Baker et al., 2025). The average ML during the fluctuation period was 486.7 kg. This value is lower than that reported by Rodriguez et al. (2025), who observed a reduction in milk yield of 712 kg per affected cow during an HPAI H5N1 outbreak in a single herd. However, both FL and ML showed considerable variation between cows (FL ranging from 10 to 113 d; ML ranging from 12.6 to 2,470 kg), indicating considerable individual variability in response to HPAI H5N1 infection and treatment.

The average parity of cows in this dataset (mean ± SD; 2.7 ± 0.9) was higher than the average parity of cows in the dataset used for the analysis of treatment necessity (mean ± SD; 1.8 ± 0.9). Given that the milk yield dataset included only cows that were treated for HPAI H5N1, this likely reflects the positive association between parity and likelihood of requiring treatment, as demonstrated in the previous analysis and in the literature (Burrough et al., 2024).

Figure 2 summarizes the estimated marginal means for the ML during the fluctuation period after HPAI H5N1 treatment across Immunity+ GEBV groups, with 95% CI. Cows with low Immunity+ GEBVs experienced significantly greater ML during the post-HPAI H5N1 treatment fluctuation period (618.10 kg; 95% CI: 561.08–675.12 kg) compared with those with average (511.45 kg; 95% CI: 484.01–538.89 kg; *P* = 0.02) or high 484.46 kg; 95% CI: 440.61–528.30 kg; *P* = 0.03) Immunity+ GEBVs. On average, cows with low Immunity+ GEBVs lost approximately 134 kg more milk during this period than cows with high Immunity+ GEBVs. Analysis of Immunity+ GEBV as a continuous variable also revealed a significant association with ML (*P* < 0.001). These results emphasize the economic advantage of selecting for higher Immunity+ GEBVs, helping producers mitigate production losses associated with emerging infectious diseases such as HPAI H5N1.

**Figure 2:**
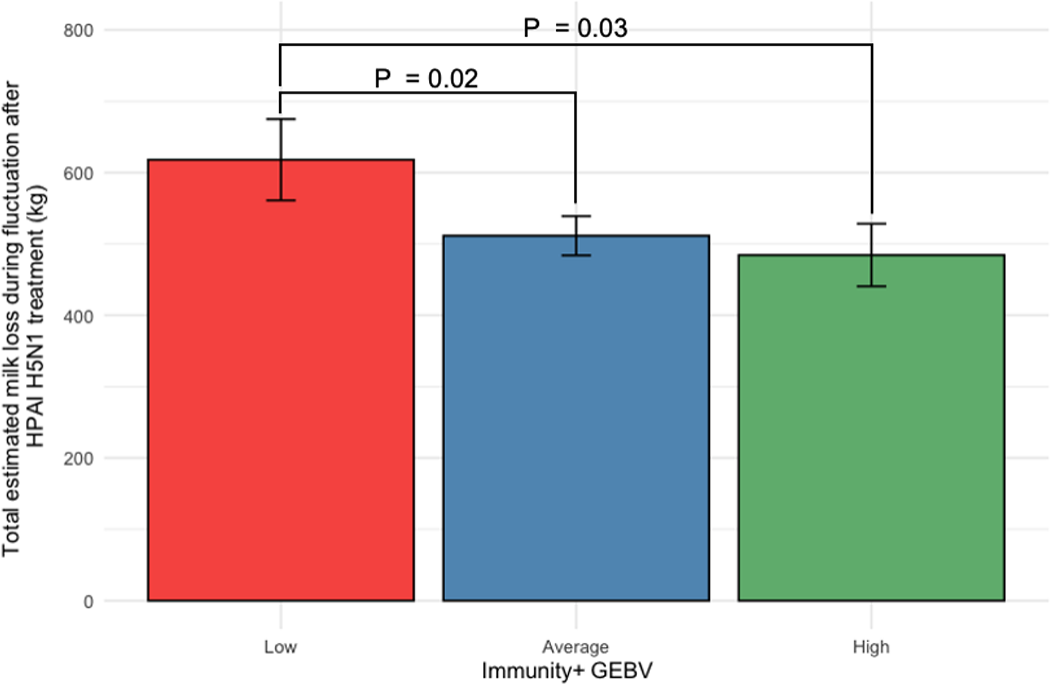
Estimated marginal means (with 95% CI) for total ML (kg) during the fluctuation period following supportive treatment for HPAI H5N1 for Immunity+ GEBV classes (low, n = 68; average n = 266; high, n = 57).

No significant difference was found in FL between Immunity+ GEBV categories. Therefore, the duration of the fluctuation period during HPAI H5N1 infection was similar regardless of Immunity+ GEBV. However, the ML experienced during this period was lower for cows with high Immunity+ GEBVs than for those with low Immunity+ GEBVs. This suggests that cows with higher Immunity+ GEBVs may be better able to maintain milk production or recover more efficiently during infection, demonstrating greater resilience in the face of disease challenge. In practical terms, this finding supports the idea that selection for improved immune response through Immunity+ can reduce the severity of production losses associated with infectious disease outbreaks, even when recovery duration remains unchanged.

Overall, the results of this study demonstrate that dairy cows with higher Immunity+ GEBVs were significantly less likely to require supportive treatment and experienced lower milk losses during HPAI H5N1 outbreaks. This reduction in treatment requirements and production losses has the potential to create considerable cost savings while supporting healthier, more resilient herds. These findings reinforce the value of selecting for enhanced immune response as part of a sustainable, long-term strategy to improve both animal health outcomes and economic resilience during disease challenges.
